# The Creosote Treatment of Tuberculosis

**Published:** 1891-09

**Authors:** Theodore Potter

**Affiliations:** Lecturer and Demonstrator in Bacteriology, Medical College of Indiana


### SELECTIONS.

### The Creosote Treatment of Tuberculosis.

BY THEODORE POTTER, A.M., M.D.

Lecturer and Demonstrator in Bacteriology, Medical College of Indiana.

In a recent number of the *Berliner Klinische Wochenschrift*, Sommerbrodt, the chief advocate of the creosote treatment of tuberculosis, gave a review of his thirteen year's experience. Various authorities had used creosote in this disease, but to Sommerbrodt, of Breslau, is due the chief credit for giving the method its present prominence. In again calling the attention of the profession to the value of the drug, Sommerbrodt suggests it as a supplement to the Koch treatment, and urges that it has the advantage of Koch's method in being applicable to all forms and stages of the disease, and in being subject to no contra-indications. He cites seventeen cases taken because they illustrate the points he wishes to emphasize. These cases were all seen by other physicians, the diagnosis well established in most by the presence of the tubercle bacillus and whose histories extend over a number of years. In most of them also climatic treatment was not available.

Sommerbrodt refers to the experiments by Koch and Fraentzel and by Cornet, to determine, if possible, the influence of creosote on the tubercle bacillus. Cornet inoculated several guinea-pigs with tuberculosis, and then gave them creosote by the stomach. No effect was discoverable for all the animals died of the disease. Sommerbrodt urges that such experiments should not condemn the creosote treatment; for, in the first place, experience has shown that inferences from experiments on animals must be transferred to man with great caution; and second, he quotes Koch himself as having stated that men are many times more susceptible to the "lymph" than guinea-pigs. Why, asks Sommerbrodt, may not the same thing be true of creosote?

Such experiments do not negative the facts of clinical experience.

Sommerbrodt suggests that an attempt should be made to cultivate the bacillus on blood serum obtained from men who had been under the creosote treatment for six or eight weeks. This has not yet been done. Guttman, however, has shown that 1 to 4,000 creosote will prevent the growth of the bacilli on culture soils.

The results of Sommerbrodt's experience with five thousand cases are, that in a considerable proportion, and that greater than under any other plan, definite recovery may be secured, provided the proper method is carried out. He emphasizes the importance of early diagnosis and treatment, and lays much stress upon four factors which, he says, have not heretofore been fully appreciated by the profession. These are :—first, the necessity of using a good quality of creosote ; second, large doses, *i. e.*, a gramme or more daily ; third, continuing the drug a long time, *i. e.*, several years in many cases, and always for some months after all symptoms have disappeared ; fourth, the medicine must always be given after the three principal meals, never on an empty, or comparatively stomach. He gives the creosote with balsam tolu, or cod liver oil, in small capsules. Many of his patients have taken from one to two grammes daily for long periods. Some of the cases reported have used it for two, three and four years, or even more. He insists also, that begun in small doses, increased gradually, and observing the rules laid down, it does not often disturb the digestion, but rather the opposite. The profession must change its views on this point, as his five thousand cases have taught.

This report is valuable as coming from a careful and skillful observer, but chiefly because it covers a long period of time, and is not based upon the deceptive experience of a few months. The treatment is simple, certainly free from any danger, does not exclude the use of any or all other agents, and it does look as though it were really curative.

The writer has used the creosote plan as fathered by Sommerbrodt for some time, and can, at least, confirm the statement that in but few cases, properly used, is it necessary to retreat to save the stomach.

